# The landscape of immune checkpoint inhibitor plus chemotherapy versus immunotherapy for advanced non‐small‐cell lung cancer: A systematic review and meta‐analysis

**DOI:** 10.1002/jcp.29371

**Published:** 2019-11-06

**Authors:** Chengdi Wang, Wenliang Qiao, Yuting Jiang, Min Zhu, Jun Shao, Tao Wang, Dan Liu, Weimin Li

**Affiliations:** ^1^ Department of Respiratory and Critical Care Medicine, West China Hospital, West China Medical School Sichuan University Chengdu China; ^2^ Department of Lung Cancer Center, Laboratory Lung Cancer, West China Hospital, West China Medical School Sichuan University Chengdu China; ^3^ Department of Obstetrics and Gynecology, West China Medical School Sichuan University Chengdu China

**Keywords:** chemotherapy, efficacy, immune checkpoint inhibitor, non‐small‐cell lung cancer, safety

## Abstract

**Background:**

Lung cancer is the leading cause of cancer‐related deaths worldwide and the prognosis remains poor. The recent introduction of the immune checkpoint inhibitor (ICI), or plus chemotherapy, both resulted in the survival benefit for patients with advanced non‐small‐cell lung cancer (NSCLC), but it remains unanswered which is superior. The current study aimed to estimate the comparative efficacy and safety of ICI‐chemotherapy versus ICI‐monotherapy in advanced NSCLC.

**Methods:**

Studies were identified by searching PubMed, Embase, and Cochrane library. The randomized controlled trials (RCTs) that ICI monotherapy or ICI plus chemotherapy compared with chemotherapy in NSCLC were included with available primary endpoints of progression‐free survival (PFS), overall survival (OS), objective response rate, or treatment‐related adverse events. A fixed‐effect or random‐effects model was adopted depending on between‐study heterogeneity.

**Results:**

A total of 20 RCTs involving 12,025 patients with NSCLC were included. Both ICI‐monotherapy and ICI‐chemotherapy resulted in significantly prolonged survival compared to chemotherapy and the former led to significantly longer PFS. The magnitude of survival benefits appeared to be greatest among those treated with pembrolizumab plus platinum‐based chemotherapy (OS, 0.56; PFS, 0.54). Additionally, OS and PFS advantages of ICI therapies were observed in patients with NSCLC with low or high programmed cell death 1 ligand 1 (PD‐L1) expression level, but not in intermediate PD‐L1 TPS.

**Conclusions:**

Pembrolizumab plus platinum‐based chemotherapy was recommended as the optimal first‐line therapy for advanced patients with NSCLC. Additionally, PD‐L1 alone is not recommended as an adequate molecular biomarker to identify eligible patients for routine clinical practice in immunotherapy.

## Graphical abstract

Both ICI‐monotherapy and ICI‐chemotherapy resulted in significantly prolonged survival compared with chemotherapy and the former led to significantly longer PFS. Pembrolizumab plus platinum‐based chemotherapy was recommended as the optimal first‐line therapy for advanced patients with NSCLC. PD‐L1 alone is not recommended as an adequate molecular biomarker to identify eligible patients for routine clinical practice in immunotherapy.

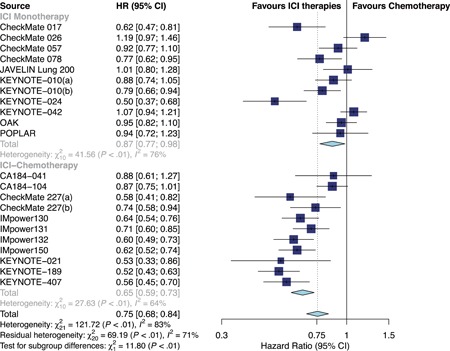

**Abbreviations**
ATE: atezolizumab
CI: confidence interval
CTLA‐4: cytotoxic T lymphocyte associated antigen 4
DOC: docetaxel
ECOG PS: Eastern Cooperative Oncology Group Performance Status
HR: hazard ratio
ICI: immune checkpoint inhibitor
IPI: ipilimumab
NIV: nivolumab
NSCLC: non‐small‐cell lung cancer
ORR: objective response rate
OS: overall survival
PBC: platinum‐based chemotherapy
PD‐1: programmed cell death 1
PD‐L1: programmed cell death 1 ligand 1
PEM: pembrolizumab
PFS: progression‐free survival
RCT: randomized controlled trial
RR: risk ratio
SITC: the Society for Immunotherapy of Cancer
TPS: tumor proportion score
TRAEs: treatment‐related adverse events
WCLC: World Conference on Lung Cancer

## 1 INTRODUCTION

Lung cancer is the leading cause of cancer death worldwide (Davies, Cheng, Field, Liu, & Li, 2019; Siegel, Miller, & Jemal, 2018). Most patients with lung cancer are diagnosed at an advanced stage with metastasis (Brahmer et al., 2018). At the metastatic stage, the 5‐year survival rate is no more than 5% since no curative treatment options. However, only a small population of patients (16%) are diagnosed at an early stage, for which the 5‐year survival rate amounts to 56% (Siegel et al., 2018). Non‐small‐cell lung cancer (NSCLC) presents as the most prevalent histological subtype (>85%) of lung cancer (Herbst, Morgensztern, & Boshoff, 2018). Squamous‐cell NSCLC, accounting for approximately 30% of all cases of NSCLC and lacking the targetable genetic aberrations, is related with worse prognosis than is nonsquamous‐cell NSCLC (Paz‐Ares et al., 2018).

The standard‐of‐care therapies as first‐line treatments for patients with advanced NSCLC include platinum‐doublet chemotherapy for those with nonsquamous cancer and targeted treatments for those with targetable genetic aberrations (Planchard et al., 2019; Sandler et al., 2006). However, the clinical outcomes of patients with NSCLC remain poor. Some reasons may explain this. On the one hand, platinum‐based chemotherapy (PBC) only provides advanced NSCLC patients with a 15–30% response rate. On the other hand, new agents, such as docetaxel (DOC), can significantly improve survival benefits comparing with supportive care, but at the expense of a higher risk of adverse events (Fillon, 2018). Furthermore, only a small proportion of patients with NSCLC could benefit from targeted therapy due to the lack of targetable mutations (Camidge, Doebele, & Kerr, 2019).

Recently, growing evidence indicates that immune checkpoint inhibitor (ICI) therapies are promising therapeutic options for patients with NSCLC. Several ICI drugs have been approved by Food and Drug Administration and they could inhibit downregulation of antitumor responses through blocking programmed death 1 (PD‐1), programmed death ligand 1 (PD‐L1), and cytotoxic T‐cell lymphocyte antigen 4 (CTLA‐4) pathway in mechanism (Ribas & Wolchok, 2018). NSCLC tumor cells escape immune attack and induce tumor tolerance through developing immune checkpoints. For example, the tumor express ligand PD‐L1, which is prevalent in NSCLC, is engaged by the activated T expressed receptor PD‐1 to downregulate the antitumor function of T cells and promote immune escape (Pardoll, 2012). By blocking such immune checkpoints, the activation of T cells could be continued and the immune system could eliminate NSCLC cancer cells (Pardoll, 2012). The anti‐PD‐1 monotherapy is the first‐line treatment for patients with NSCLC with at least 50% or more of PD‐L1 expression on tumor cells and platinum‐doublet chemotherapy plus anti PD‐1 is the first‐line therapeutic option for nonsquamous‐cell NSCLC. ICIs, including anti‐PD‐1, anti‐PD‐L1, and anti‐CTLA‐4 antibodies, have shown great promising benefit in clinical trials and have been rapidly incorporated into the standard management of advanced stage NSCLC. Treatment with ICIs in patients with advanced NSCLC has achieved landmark 3‐year overall survival (OS) rates of 19% and 26.4% among previously‐treated and treatment‐naive patients, respectively, and up to more than 18 months of progression‐free survival (PFS; Remon, Reguart, Auclin, & Besse, 2019).

However, the relative efficacy and safety of different ICI strategies for advanced patients with NSCLC remains controversy. In clinical practice, current ICI strategies contain two or three of the following treatment or different doses of the same ICI drug, including nivolumab (NIV), pembrolizumab (PEM), avelumab, atezolizumab (ATE), ipilimumab (IPI), and conventional therapy (chemotherapy or and targeted therapy). Furthermore, it is hypothesized that ICI plus chemotherapy (ICI‐chemotherapy) might exhibit synergistical effects on survival benefits because the antitumor activity could be mediated by the cytotoxic effects of chemotherapy and the immunological effects of ICI therapies (e.g., chemotherapy could induce PD‐L1 expression on tumor cells and infiltrating immune cells therefore enhancing the therapeutic effects of ICI therapies; Havel, Chowell, & Chan, 2019). ICI therapies, harnessing the immune system, are demonstrating promising results in combination with chemotherapy (Socinski et al., 2018). Nevertheless, in a Phase III randomized controlled trial (RCT), ATE plus PBC failed to show any benefits over chemotherapy with respect to OS (Jotte et al., 2018). More important, there is no head‐to‐head study concerning comparison of ICI‐chemotherapy versus ICI monotherapy to validate their comparative efficacy and safety.

Therefore, the appropriate treatment algorithm of ICI therapies for advanced patients with NSCLC remains an unmet need. This study aimed to explore whether ICI monotherapy or ICI‐chemotherapy could lead to better efficacy and more manageable side‐effect profile than chemotherapy in advanced patients with NSCLC and to further investigate whether the benefits and risks of ICI‐monotherapy would differ from ICI‐chemotherapy through an indirect comparison. We expected that our results with evidence from 20 Phase II/III RCTs involving 12,025 patients could contribute to the development of ICI therapies or plus chemotherapy and provide practical suggestions on the routine clinical practice of ICI therapies for advanced patients with NSCLC.

## 2 MATERIALS AND METHODS

### 2.1 Search strategy and study selection

We searched for eligible RCTs from PubMed, Embase, and the Cochrane Central Register of Controlled Trials from January 2010 to July 2019. The keywords included ICIs (anti PD‐1 or anti PD‐L1 or anti CTLA‐4), specific ICI drug names (avelumab, ATE, durvalumab, IPI, NIV, PEM, tremelimumab), and lung cancer (The search strategy is detailed in Table S1). We retrieved additional studies from major conference proceedings of the American Society of Clinical Oncology, the European Society of Medical Oncology, the American Association for Cancer Research, and the World Conference on Lung Cancer (WCLC). In terms of duplicated studies, the most complete data of the study was enrolled.

Study selection corresponded with the Preferred Reporting Items for Systematic Reviews and Meta‐Analyses statement (Knobloch, Yoon, & Vogt, 2011; Liberati et al., 2009). Both exclusion and inclusion criteria were prespecified. The eligible RCTs met the following inclusion criteria as follow: (a) population: pathologically confirmed advanced patients with NSCLC; (b) intervention: treated with PD‐1/PD‐L1/CTLA‐4 inhibitors (avelumab, ATE, durvalumab, IPI, NIV, PEM, or tremelimumab) with or without chemotherapy irrespective of dosage and duration; (c) comparison: treated with chemotherapy; (d) outcomes: PFS or OS measured as hazard ratios (HRs), objective response rate (ORR), and treatment‐related adverse events (TRAEs) of any grade or grade ≥3 measured as risk ratios (RR). Studies were excluded based on the following criteria as follows: (a) designed as retrospective or prospective observational cohort studies; (b) lack of related data; (c) published as reviews, case reports, letters, commentaries, editorials, or meta‐analysis; and (d) duplicated articles. Manual search was performed through reviewing the reference lists of all trials fulfilling the eligibility criteria for additional relevant studies.

### 2.2 Data extraction and quality assessment

The following items were extracted for each trial: first author, year of publication, acronym of the trial, trial phase, histology type, number of patients, OS, PFS, ORR, and TRAEs of any grade and grade ≥3. We carried out the methodological quality assessment of the enrolled trials with the Cochrane Risk of Bias Tool (Higgins et al., 2011), which consists of six items: random sequence generation; allocation concealment; blinding of participants and personnel to the study protocol; blinding of outcome assessment; incomplete outcome data; and selective reporting. An item identified as “low risk” is considered as applicable. Two authors (W. Q. and Y. J.) independently extracted data and performed quality assessment in this process and discrepancies were resolved by consensus (C. W.).

### 2.3 Statistical analysis

The *χ* ^2^ test and *I* ^2^ statistic were applied to evaluate heterogeneity. The random effect models were chosen if *I* ^2^ was more than 50%, implying obvious heterogeneity, otherwise, the fixed‐effect models were applied (Liberati et al., 2009). The primary outcomes were OS and PFS, presented with HRs, 95% CIs, and *p* values, which were calculated using the inverse‐variance‐weighted method. The integrated analysis for ORR, Grade 1–5 TRAEs, and Grade 3–5 TRAEs were conducted based on the Mantel–Haenszel method. The Bucher's method was employed to make each of the pairwise indirect comparisons separately (Sultan, 2009). Subgroup analysis was conducted to explore the potential source of heterogeneity. The publication bias of the enrolled studies was assessed by Begg's and Egger's tests (Egger, Davey Smith, Schneider, & Minder, 1997). All analyses were performed by using the Stata 15.0 software (Stata Corp, College Station, TX). Two‐sided *p* < .05 was considered statistically significant.

## 3 RESULT

### 3.1 Literature search

A total of 3,691 related studies were identified by the initial search strategy (Figure S1). Finally, 20 randomized controlled trails with 22 studies involving 12,025 patients were included for the quantitative analysis (Barlesi et al., 2018; Borghaei et al., 2015; Borghaei et al., 2018 2018; Brahmer et al., 2015; Carbone et al., 2017; Fehrenbacher et al., 2016; Gandhi et al., 2018; Govindan et al., 2017; Hellmann et al., 2018; Herbst et al., 2016; Jotte et al., 2018; Langer et al., 2016; Lynch et al., 2012; Mok et al., 2019; Papadimitrakopoulou et al., 2018; Paz‐Ares et al., 2018; Reck et al., 2016; Rittmeyer et al., 2017; Socinski et al., 2018; West et al., 2019; Wu et al., 2019). Half of these 22 studies investigated the efficacy of ICI monotherapy versus chemotherapy, including 1 study about avelumab, 2 about ATE, 4 about NIV, and 4 about PEM. Furthermore, the remaining 11 studies compared the cooperative efficacy of ICI‐chemotherapy compared with chemotherapy. Figure S1 demonstrates the algorithm to identify inclusion articles. Data were retrieved from publications of eligible studies while IMpower131 and IMpower132 could only be obtained from the American Society of Clinical Oncology and WCLC presentations.

### 3.2 Characteristics of the studies and quality assessment

All RCTs were international multicenter studies published between 2012 and 2019, which were funded by the pharmaceutical industry. All trails were completed in advanced or metastatic settings including Stage IIIB or IV or recurrent patients with NSCLC, who had the Eastern Cooperative Oncology Group performance‐status (ECOG PS) score of 0 or 1. 17 of 20 eligible trials belonged to Phase III studies, and POPLAR and CA184‐041 were Phase II trials. There were 6,490 patients enrolled in the intervention group (ICI‐chemotherapy or ICI‐monotherapy) and 5,535 patients allocated to the chemotherapy control group. Among patients in the intervention arm, 53.5% were treated with ICI monotherapy compared with chemotherapy and 46.5% were treated with ICI‐chemotherapy compared with chemotherapy. Four studies were conducted with squamous lung cancer, six with nonsquamous lung cancer, and 12 with mixed types of squamous and nonsquamous cancer. Overall, all but seven studies (35%) demonstrated an OS advantage for patients receiving ICI therapies compared with patients receiving chemotherapy. In subgroup analyses, eight studies (40%) showed an OS advantage from ICI monotherapy and five studies (25%) showed the same advantage from ICI‐chemotherapy compared with chemotherapy.

Two RCTs had unique designs and were needed to warrant further explanation. KEYNOTE‐010 was a Phase II/III trial and evaluated two different doses of PEM (2 mg/kg and 10 mg/kg) every 3 weeks, which was considered as two studies—(a) KEYNOTE‐010 and (b) KEYNOTE‐010. CheckMate 227 trial explored the efficacy of two NIV‐based treatments (NIV plus IPI or NIV) with an additional PBC compared with chemotherapy, which was descried as (a) CheckMate 227 and (b) CheckMate 227 in this study. The main characteristics of the eligible RCTs are shown in Table 1 and the quality of these trails was satisfactory (Table S2). Sensitivity analysis illustrated our results remained stable by omitting trails sequentially (Figure S2 and S3) and the funnel plot revealed no evidence of publication bias (Figure S4).

**Table 1:** Clinicopathological characteristics of the included randomized controlled trials

Author	Year	Study ID	Disease stage	Trial phase	Histology type	No. of patients	Treatment	ORR	OS (95%)	PFS (95%)	Grade 1–5 TRAE	Grade 3–5 TRAEs
**ICI monotherapy**												
Brahmer	2015	CheckMate 017	IIIB,	III	Squamous	135	NIV	27 (135)	0.59 (0.44–0.79)	0.62 (0.47–0.81)	76 (131)	9 (131)
			IV			137	DOC	12 (137)			111 (129)	71 (129)
Carbone	2017	CheckMate 026	IV,	III	Squamous,	271	NIV	55 (211)	1.08 (0.87–1.34)	1.19 (0.97–1.46)	190 (267)	47 (267)
			Recurrent		Nonsquamous	270	PC	71 (212)			243 (263)	133 (263)
Borghaei	2015	CheckMate 057	IIIB, IV	III	Nonsquamous	292	NIV	56 (292)	0.73 (0.59–0.89)	0.92 (0.77–1.11)	199 (287)	30 (287)
			Recurrent			290	DOC	36 (290)			236 (268)	144 (268)
Wu	2019	CheckMate 078	IIIB, IV	III	Squamous,	338	NIV	56 (338)	0.68 (0.52–0.90)	0.77 (0.62–0.95)	216 (337)	35 (337)
			Recurrent		Nonsquamous	166	DOC	7 (166)			130 (156)	74 (156)
Barlesi	2018	JAVELIN Lung 200	IIIB, IV	III	Squamous,	396	AVE	NR	0.90 (0.75–1.08)	1.01 (0.80–1.28)	251 (393)	39 (393)
			Recurrent		Nonsquamous	396	DOC				254 (365)	180 (365)
Herbst	2016	KEYNOTE‐010 (a)	IIIB, IV	II/III	Squamous,	344	PEM (2 mg/kg)	62 (344)	0.71 (0.58–0.88)	0.88 (0.74–1.05)	215 (339)	43 (339)
			Recurrent		Nonsquamous	172	DOC	16 (172)			126 (155)	55 (155)
Herbst	2016	KEYNOTE‐010 (b)	IIIB, IV	II/III	Squamous,	346	PEM (10 mg/kg)	64 (346)	0.61 (0.49–0.75	0.79 (0.66–0.94)	226 (343)	55 (343)
			Recurrent		Nonsquamous	171	DOC	16 (171)			125 (154)	54 (154)
Reck	2016	KEYNOTE‐024	IV	III	Squamous,	154	PEM	69 (154)	0.60 (0.41–0.89)	0.50 (0.37–0.68)	113 (154)	41 (113)
					Nonsquamous	151	PC	42 (151)			135 (150)	80 (150)
MOK	2019	KETNOTE‐042	IIIB, IV	III	Squamous,	637	PEM	174 (637)	0.81 (0.71–0.93)	1.07 (0.94–1.21)	399 (636)	113 (636)
					Nonsquamous	637	PC	169 (637)			553 (615)	252 (615)
Rittmeyer	2016	OAK	IIIB, IV	III	Squamous,	425	ATE	58 (425)	0.73 (0.62–0.87)	0.95 (0.82–1.10)	390 (609)	90 (609)
					Nonsquamous	425	DOC	57 (425)			496 (578)	247 (578)
Fehrenbacher	2016	POPLAR	IIIB, IV	II	Squamous,	144	ATE	21 (144)	0.73 (0.53–0.99)	0.94 (0.72–1.23)	95 (142)	57 (142)
					Nonsquamous	143	DOC	21 (143)			119 (135)	71 (135)
ICI‐Chemotherapy												
Lynch	2012	CA184‐041	IIIB, IV	II	Squamous,	70	IPI plus PBC	15 (70)	0.99 (0.67–1.46)	0.88 (0.61–1.27)	54 (71)	29 (71)
			Recurrent		Nonsquamous	66	PBC	9 (66)			52 (65)	24 (65)
Govindan	2017	CA184‐104	IV,	III	Squamous	388	IPI plus PBC	171 (388)	0.91 (0.77–1.07)	0.87 (0.75–1.01)	344 (388)	205 (388)
			Recurrent			361	PBC	170 (361)			292 (361)	129 (361)
Hellmann	2018	CheckMate 227(a)	IV,	III	Squamous,	139	NIV, IPI plus PBC	63 (139)	NR	0.58(0.41–0.81)	433 (576)	180 (576)
			Recurrent		Nonsquamous	160	PBC	43 (160)			460 (570)	206 (570)
Barghaei	2018	CheckMate 227(b)	IV,	III	Squamous,	177	NIV plus PBC	65 (177)	NR	0.74 (0.58–0.94)	158 (172)	89 (172)
			Recurrent		Nonsquamous	186	PBC	43 (186)			141 (183)	64 (183)
West	2019	IMpower130	IV	III	Nonsquamous	451	ATE plus PBC	220 (447)	0.79 (0.64–0.98)	0.64 (0.54–0.77)	455 (473)	354 (473)
						228	PBC	72 (226)			215 (232)	141 (232)
Jotte	2018	IMpower131	IV,	III	Squamous	343	ATE plus PBC	169 (343)	0.96 (0.78–1.18)	0.71 (0.60‐0.85)	316 (334)	231 (334)
			Recurrent			340	PBC	140 (340)			303 (334)	193 (334)
Papadimitrakopoulou	2018	IMpower132	IV	III	Nonsquamous	292	ATE plus PBC	137 (292)	0.81 (0.64–1.03)	0.60 (0.49–0.72)	267 (291)	167 (291)
						286	PBC	92 (286)			239 (274)	114 (274)
Socinski	2018	IMpower150	IV,	III	Nonsquamous	400	ATE plus PBC	224 (353)	0.78 (0.64–0.96)	0.62 (0.52–0.74)	371 (393)	230 (393)
			Recurrent			400	PBC	159 (331)			376 (394)	197 (394)
Langer	2016	KEYNOTE‐021	IIB,	III	Nonsquamous	60	PEM plus PBC	33 (60)	0.56 (0.32–0.95)	0.53 (0.33–0.86)	55 (59)	24 (59)
			IV			63	PBC	18 (63)			57 (62)	17 (62)
Gandhi	2018	KEYNOTE‐189	IV	III	Nonsquamous	410	PEM plus PBC	195 (410)	0.49 (0.38–0.64)	0.52 (0.43–0.64)	404 (405)	272 (405)
						206	PBC	39 (206)			200 (202)	133 (202)
Paz‐Ares	2018	KEYNOTE‐407	IV	III	Squamous	278	PEM plus PBC	161 (278)	0.64 (0.49–0.85)	0.56 (0.45–0.70)	273 (278)	194 (278)
						281	PBC	108 (281)			274 (280)	191 (280)

Abbreviations: ATE, atezolizumab; AVE, avelumab; DOC, docetaxel; TRAE, treatment‐related adverse event; ICI, immune checkpoint inhibitor; IPI ipilimumab; NIV nivolumab; ORR, objective response rate; OS, overall survival; PBC, platinum‐based chemotherapy; PEM, pembrolizumab; PFS, progression‐free survival.

### 3.3 Primary analysis

#### 3.3.1 Efficacy

**Figure 1:**
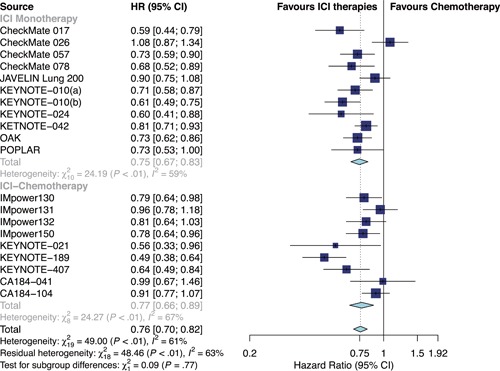
Forest plots of HRs comparing overall survival between ICI‐chemotherapy and ICI monotherapy. CI, confidence interval; HR hazard ratio; ICI, immune checkpoint inhibitor

**Figure 2:**
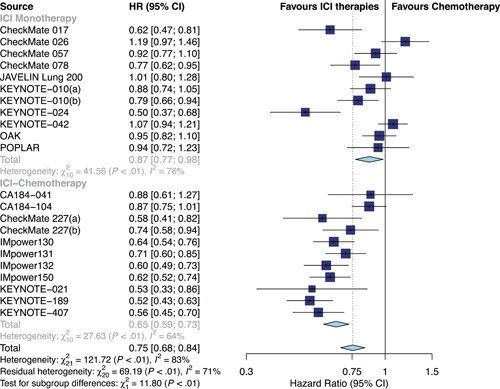
Forest plots of HRs comparing progression‐free survival between ICI‐chemotherapy and ICI‐monotherapy. CI, confidence interval; HR hazard ratio; ICI, immune checkpoint inhibitor

**Figure 3:**
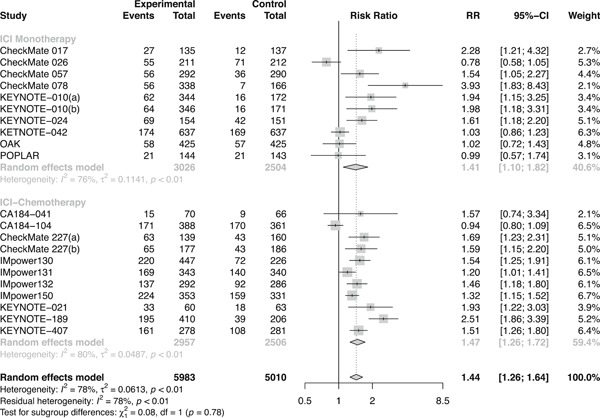
Forest plots of RRs comparing objective response rate between ICI‐chemotherapy and ICI‐monotherapy. CI, confidence interval; ICI, immune checkpoint inhibitor; RR, risk ratio

**Figure 4:**
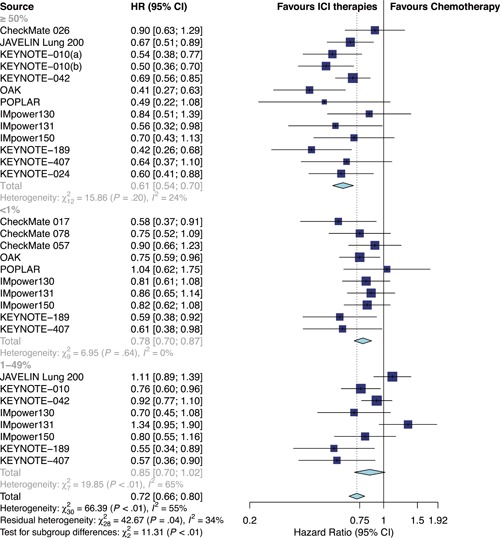
Forest plots of HRs comparing overall survival between ICI therapies and chemotherapy according to PD‐L1 status. CI, confidence interval; HR hazard ratio; ICI, immune checkpoint inhibitor; PD‐L1, programmed cell death 1 ligand 1

Overall, ICI therapies, compared with the control group of chemotherapy, significantly reduced the risk of death (HR, 0.76; 95% confidence interval [CI], 0.70–0.82) (Figure 1) and the disease progression (HR, 0.75; 95% CI, 0.68–0.84; Figure 2). ICI therapies yielded significantly higher ORR than chemotherapy (RR, 1.44; 95% CI, 1.26–1.64; Figure 3). Besides, ICI‐chemotherapy resulted in greater PFS benefit than ICI monotherapy with statistical difference compared with chemotherapy ([HR = 0.65; 95% CI, 0.59–0.73] and [HR = 0.87; 95% CI, 0.77–0.98]; difference *p* < .01; Figure 2). Conversely, equal OS and ORR benefits were observed evenly in both ICI‐chemotherapy and ICI‐monotherapy compared with chemotherapy ([HR = 0.77; 95% CI, 0.66–0.89] and [HR = 0.75; 95% CI, 0.67–0.83]; difference *p* = .77; Figure 1; [RR = 1.47; 95% CI, 1.26–1.72] and [RR = 1.41; 95% CI, 1.10–1.82]; difference *p* = .78; Figure 3). The risk of death and the disease progression were remarkably reduced among patients who received ICI therapies compared with chemotherapy and had a high PD‐L1 expression level (OS: HR, 0.61; 95% CI, 0.54–0.70; Figure 4; PFS: HR, 0.56; 95% CI, 0.47–0.66; Figure S5). Interestingly, in patients with negative PD‐L1 level, the OS and PFS were in favor of ICI therapies (OS: HR, 0.78; 95% CI, 0.70–0.87; PFS: HR, 0.77; 95% CI, 0.67–0.89). However, there was no survival advantage of ICI therapies over chemotherapy for patients with intermediate PD‐L1 level (OS: HR, 0.85; 95% CI, 0.70–1.02; PFS: HR, 0.76; 95% CI, 0.56–1.04). The HR difference of survival among the three subgroups including patients that had a PD‐L1 tumor proportion score (TPS) <1%, 1–49%, and TPS ≥50%, was statistically significant (OS: difference *p* < 0.01; PFS; difference *p* = .01). In terms of histology type, all patients treated with ICI therapies had superior OS (squamous: HR, 0.79; *p* < .001; nonsquamous: HR, 0.77; *p* < .001) and PFS (squamous: HR, 0.69; *p* < .001; nonsquamous: HR, 0.73; *p* < .001) compared to those treated with chemotherapy. The ORR benefit of squamous lung cancer patients using ICI therapies was marginal (*p* = .073), while there was a significant improvement of ORR in the subgroup of nonsquamous NSCLC compared with chemotherapy (*p* < .001; Figure S6).

#### 3.3.2 Safety

**Figure 5:**
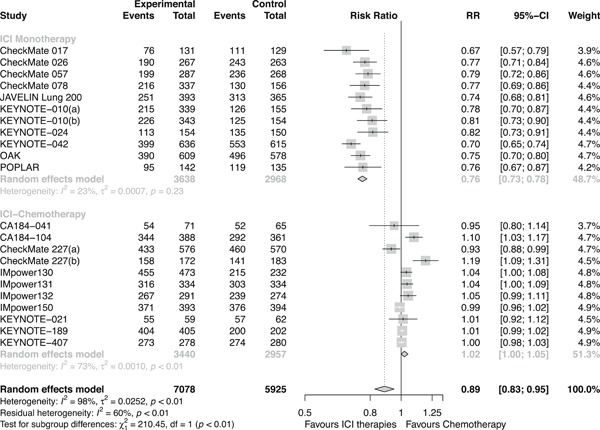
Forest plots of RRs comparing Grade 1–5 TRAE between ICI‐chemotherapy and ICI‐monotherapy. CI, confidence interval; ICI, immune checkpoint inhibitor; RR, risk ratio; TRAE, treatment‐related adverse event

**Figure 6:**
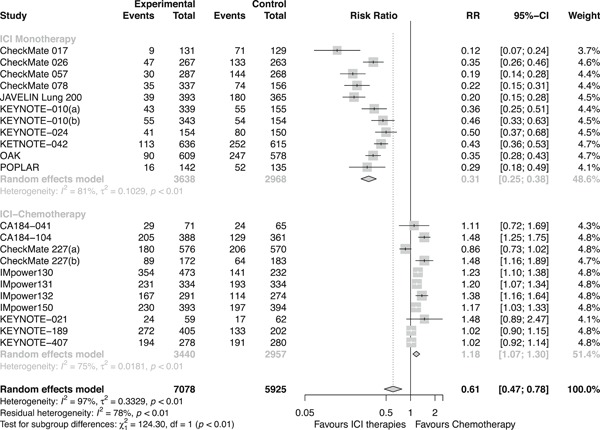
Forest plots of RRs comparing Grade 3–5 TRAEs between ICI‐chemotherapy and ICI‐monotherapy. ICI, immune checkpoint inhibitor; RR, risk ratio; TRAE, treatment‐related adverse event

Compared with chemotherapy alone, reductions of risk in favor of ICI therapies were achieved for Grade 1–5 TRAEs (RR, 0.89; 95% CI, 0.83–0.95) and Grade 3–5 TRAEs (RR, 0.61; 95% CI, 0.47–0.78; Figures 5 and 6). Regarding risk for Grade 3–5 TRAEs, compared with chemotherapy, patients receiving ICI‐chemotherapy were at higher risk than ICI‐monotherapy ([RR = 1.18; 95%,CI, 1.07–1.30] and [RR = 0.31; 95% CI, 0.28–0.38], respectively; difference *p* < .01). In terms of Grade 1–5 TRAEs, compared with chemotherapy, patients receiving ICI‐chemotherapy were also at higher risk than ICI‐monotherapy ([RR = 1.02; 95% CI, 1.00–1.05] and [RR = 0.76; 95% CI, 0.73–0.78], respectively; difference *p* < .01).

### 3.4 Subgroup analysis

#### 3.4.1 ICI‐chemotherapy vs ICI monotherapy

Table 2 and Table S3 revealed the differences in efficacy of ICI‐chemotherapy and ICI‐monotherapy in the subgroups. Noticeably, the magnitude of OS benefit was greater in patients treated with anti‐PD‐1 blocker plus chemotherapy than those receiving anti‐PD‐1 monotherapy compared with chemotherapy ([HR, 0.56; 95% CI, 0.47–0.67] and [HR, 0.72; 95% CI, 0.64–0.82], respectively, difference *p* = .02), which was not detected in anti‐PD‐L1 therapy (anti‐PD‐L1 blocker plus chemotherapy: HR, 0.83; anti‐PD‐L1 blocker monotherapy: HR, 0.81; difference *p* = .67). In nonsmoker, ICI‐chemotherapy revealed an OS advantage over ICI monotherapy ([HR, 0.36; 95% CI, 0.15–0.85] and [HR, 1.01; 95% CI, 0.79–1.29], respectively; difference *p* = .02). In ECOG PS score of 1, both ICI‐chemotherapy and ICI monotherapy had the OS benefit compared with chemotherapy ([HR, 0.77; 95% CI, 0.70–0.85] and [HR, 0.76; 95% CI, 0.69–0.83], respectively; difference *p* = .002). However, we further conducted several subgroup analyses according to sex, age, histological type, PD‐L1 status, smoking status, and ECOG PS score of 0. No statistically significant differences in the OS benefit were found between ICI‐chemotherapy and ICI monotherapy in these subgroup analyses.

**Table 2:** Differences in PFS benefits of ICI‐chemotherapy and ICI monotherapy by subgroups

Variable	Study	Pooled HR (95% CI)		Test for difference	
		ICI‐chemotherapy	ICI‐monotherapy	*I* ^2^ (%)	*p* Value
Overall	22	0.65 (0.59; 0.73)	0.87 (0.77; 0.98)	92	<.01
Sex					
Male	13	0.66 (0.59; 0.73)	0.70 (0.58; 0.85)	0	.56
Female	13	0.54 (0.45; 0.66)	0.99 (0.75; 1.30)	92	<.01
Age (year)					
≥65	14	0.63 (0.56; 0.71)	0.82 (0.63; 1.07)	68	.08
<65	13	0.58 (0.49; 0.69)	0.85 (0.71; 1.01)	89	<.01
Smoking status					
Nonsmoker	8	0.57 (0.42; 0.76)	1.81 (1.27; 2.57)	96	<.01
Ever smoker	10	0.62 (0.57; 0.68)	0.79 (0.64; 0.99)	75	.04
First line	13	0.65 (0.59; 0.73)	0.77 (0.33; 1.81)	0	.70
Histological type					
Squamous	10	0.70 (0.57; 0.87)	0.68 (0.55; 0.85)	0	.86
Nonsquamous	12	0.59 (0.54; 0.65)	0.95 (0.74; 1.21)	92	<.01
Class of immunotherapy					
Anti‐PD‐1	12	0.59 (0.49; 0.69)	0.83 (0.71; 0.98)	88	<.01
Anti‐PD‐L1	7	0.64 (0.59; 0.70)	0.96 (0.86; 1.08)	97	<.001
PD‐L1 Status					
<1%	12	0.70 (0.62; 0.79)	0.99 (0.80; 1.23)	86	<.01
1–49%	9	0.63 (0.55; 0.72)	1.32 (0.82; 2.14)	88	<.01
≥50%	15	0.41 (0.34; 0.49)	0.66 (0.57; 0.77)	94	<.01
ECOG PS					
0	12	0.57 (0.50; 0.65)	0.86 (0.61; 1.22)	78	.03
1	12	0.74 (0.64; 0.87)	0.76 (0.63; 0.91)	0	.88

Abbreviations: CI, confidence interval; ECOG PS, Eastern Cooperative Oncology Group Performance Status; HR, hazard ratio; ICI, immune checkpoint inhibitor; PD‐1, programmed cell death 1; PD‐L1, programmed cell death 1 ligand 1; PFS, progression‐free survival.

Unlike what was observed regarding OS, for subgroups including female, young patients, patients with nonsmoker and nonsquamous‐lung cancer, and ICI‐chemotherapy was associated with prolonged PFS compared with ICI monotherapy (all difference *p* < .01). Moreover, compared with ICI monotherapy, ICI‐chemotherapy resulted in greater PFS benefit relatively to ICI monotherapy in patients who had a negative (ICI‐chemotherapy: HR, 0.70; ICI monotherapy: HR = 0.99; difference *p* < .01), intermediate (ICI‐chemotherapy: HR, 0.63; ICI monotherapy: HR, 1.32 difference *p* < .01) or high PD‐L1 expression level (ICI‐chemotherapy: HR, 0.41; ICI monotherapy: HR, 0.66; difference *p* < .01). Anti‐PD‐1 blocker plus chemotherapy was more likely to reduce the risk of disease progression than anti‐PD‐1 blocker monotherapy (HR, 0.59 and HR, 0.83, respectively; difference *p* < .01). A similar effect was found in anti‐PD‐L1 blocker (anti‐PD‐L1 blocker plus chemotherapy: HR, 0.64; anti‐PD‐L1 blocker monotherapy: HR, 0.96, difference *p* < .01). Nevertheless, PFS benefit from ICI‐chemotherapy was equivalent to that from ICI monotherapy without significant differences in the two groups for male, ever smoker, old or squamous patients with NSCLC.

#### 3.4.2 PEM plus PBC versus PEM monotherapy

The greatest improvement for OS (HR, 0.56) and PFS (HR, 0.59) was achieved in patients treated with anti‐PD‐1 blocker plus chemotherapy. Moreover, the subgroup analysis revealed that PEM plus PBC maximized the survival benefits in the ICI‐chemotherapy subgroup (OS: HR, 0.56; PFS, HR, 0.54; Figure S6). And in the ICI monotherapy subgroup, the minimum risk of death or disease progression was detected in PEM (OS: HR, 0.70; PFS, HR, 0.72; Figure S6). Thus, we further indirectly compared PEM plus PBC and PEM monotherapy as first‐line treatment with the same control group of PBC. Indirect compassion revealed that PEM plus PBC decreased the risk of death by 39% for patients with PD‐L1 TPS 1–49% (OS: HR, 0.61; 95% CI, 0.42–0.89, *p* = .01), compared with PEM monotherapy (Figure S7). For patients with PD‐L1 TPS ≥50%, although there was no significant difference in OS (HR, 0.74; 95% CI, 0.49–1.12, *p* = .15) between the two intervention groups, our indirect analysis indicated that PEM plus PBC was correlated with longer PFS than PEM monotherapy (HR, 0.51; 95% CI, 0.37–0.72, *p* < .001; Figure S7).

## 4 DISCUSSION

The pooled analysis, including 20 RCTs of high quality involving 12,025 patients, revealed that ICI therapies were associated with significantly better therapeutic effect across all the efficacy and safety end points than chemotherapy alone in advanced NSCLC populations. Compared with chemotherapy, ICI‐chemotherapy resulted in significantly prolonged PFS than ICI monotherapy with significant difference between the two subgroups. Furthermore, PEM plus PBC led to the greatest improvement for OS and PFS than the other treatments, and consequently it is recommended as the optimal first‐line option for advanced patients with NSCLC.

The current research also revealed that the magnitude of treatment effects of ICI therapies was associated with the type of cancer histology. ICI therapies decreased the risk of death by 23% in patients with nonsquamous‐cell NSCLC, which was associated with 1.63 times the possibility of achieving ORR and with comparably higher safety than stand‐of‐care therapy. However, this study indicated that ICI therapies merely correlated with a mere 21% reduction in the risk of death for squamous‐cell patients with NSCLC without improvement in ORR, which indicated that those with squamous NSCLC received less therapeutic effects than those with nonsquamous NSCLC. In general, treatment for patients with squamous NSCLC has been an area of unmet need with the little improvement of therapeutic effects since the approval of DOC in 1999 (Fillon, 2018; Melosky et al., 2016). By contrast, this study demonstrated that anti‐PD‐1 therapy seemed to be a more efficacious treatment choice than anti‐PD‐L1 therapy among squamous‐cell patients with NSCLC. There are several potential explanations for the promising clinical effects of anti‐PD‐1 antibodies. First, it is well established that tobacco smoking precipitates squamous carcinogenesis and is primarily responsible for the mutagenesis, which influences DNA repair and replication (Rizvi et al., 2015). This is in accordance with the observation that nonsynonymous mutation burden is elevated in squamous‐cell NSCLC patients (CheckMate 227, a Phase III RCT, focused on patients with NSCLC with a high tumor mutational burden). Then certain somatic mutations increase the burden of neoantigens, which is crucial for the clinical response of PD‐1 inhibitors against tumor (Łuksza et al., 2017; Rizvi et al., 2015). Consequently, nonsynonymous mutation burden strongly induces reactivity of T‐cell and results in tumor regression in the context of anti‐PD‐1 therapy (Le et al., 2017; Riaz et al., 2017; Rizvi et al., 2015).

We found that the anti‐PD‐1 therapy appeared to illicit greater treatment benefits compared with the anti‐PD‐L1 therapy, which is consistent with the previous hypothesis that NSCLC patients with anti‐PD‐1 therapy are more likely to experience prolonged survival and a more tolerable safety prolife than anti‐PD‐L1 therapy (Brahmer et al., 2018; Wei et al., 2017; Xu et al., 2018). Theoretically, the interaction between PD‐1 and PD‐L1 as the dominant ligand, can be inhibited by both PD‐L1 blockers and PD‐1 blockers. In addition, PD‐1 rather than PD‐L1 inhibitors can also block the binding of PD‐1 to PD‐L2, which is 2–6 folds stronger than the affinity of PD‐1 binding to PD‐L1(Ribas & Wolchok, 2018). PD‐L1 immunotherapy spares the mutual effects between PD‐L1 and PD‐L2. However, few RCTs are reported to have specifically investigated the role of PD‐L2 in the immunotherapy compared with conventional therapy. Further research are needed to understand the role of activation of PD‐1 pathway in the antitumor immunity on the whole landscape.

ICI‐chemotherapy and ICI monotherapy have never been directly compared in RCTs, partially because sponsors of RCTs are competitive pharmaceutical enterprises. In the presence of statistically significant interaction between ICI‐chemotherapy and ICI monotherapy, the current study revealed that there were greater PFS benefits from ICI‐chemotherapy among subgroups involving woman, young (<65 years old) patients, never smokers and nonsquamous‐lung cancer patients compared with those from ICI‐monotherapy. Our finding further confirmed that anti‐PD‐1 blocker plus chemotherapy provided OS and PFS advantages over anti‐PD‐1 therapy alone with significant difference between the two intervention groups. Previous molecular interactions aside, the observation of synergetic effects in patients with NSCLC treated with anti‐PD‐1 blocker plus chemotherapy seemed to accord with the hypothesis that chemotherapy may upregulate PD‐L1 expression level as well as promote antitumor immunity. Of particular note, PEM plus chemotherapy demonstrated the greatest benefits across all the efficacy end points compared with the other treatments. The study obtained unique findings through further indirectly comparing PEM plus PBC versus PEM alone as first‐line treatment. With the same control group of PBC, results of indirect comparison indicated that PEM plus PBC significantly improved PFS by 49% and showed numerically better OS for patients with NSCLC with PD‐L1 TPS ≥50% than PEM alone. Thus, it seems reasonable to recommend PEM plus PBC as the best treatment for patients with advanced, nonsquamous‐cell NSCLC and no actionable targeted genetic mutations. This is consistent with the recommendation of the Task Force of the Society for Immunotherapy of Cancer (SITC; Brahmer et al., 2018).

In addition, there is an unmet need for therapeutic options for advanced squamous‐cell NSCLC patients because current cytotoxic chemotherapy lacks efficacy or exerts toxicity, or because this historical subtype lacks targetable genetic mutations that targeted treatment depends upon (Carlisle & Ramalingam, 2019). By contract, this study suggests that PEM plus PBC is a preferable strategy for the squamous NSCLC group regardless of PD‐L1 status, while the SITC recommended PEM alone for squamous patients with NSCLC with PD‐L1 ≥50%. This difference can be explained as follows. The randomized Phase III study (KEYNOTE‐407) reported therapeutic effects of combination PEM plus PBC versus chemotherapy alone for squamous patients with NSCLC (Paz‐Ares et al., 2018). SITC did not take this RCT (KEYNOTE‐407) into account (Brahmer et al., 2018), because the Biologics License Application regarding with PEM plus chemotherapy for patients with squamous NSCLC was under review as of SITC's recommendation in 2018. Thus, we recommended PEM plus PBC as the optimal treatment approach for squamous patients with NSCLC.

### 4.1 Implications of the study

Our research has two main clinical implications. One is the recommended treatment strategy for NSCLC populations. As the use of ICI therapies grows, nononcology specialists of pneumology department will be increasingly called upon to decide which is the optimal treatment of the various ICI therapies for patients. In this study, we suggest PEM plus PBC as the optimal first‐line treatment for patients with advanced NSCLC but without actionable mutations (ALK, EGFR, ROS1).

Another implication of our research is that PD‐L1 alone does not seem to be an ideal predictive biomarker for clinical outcomes of patients with NSCLC treated with ICI. Most of patients with NSCLC are diagnosed at an advanced stage (e.g., with metastasis), which indicates the limited scope for curative treatment. Thus, it is increasingly crucial to identify sensitive and specific biomarkers of clinical responses to ICI therapies so as to select eligible patients for immunotherapy. PD‐L1 expression status is one of the most researched markers, and an important unanswered question in this field is whether PD‐L1 expression level is an adequate predictive biomarker for tumor response. In the present study, significantly improved OS and PFS were observed in patients with NSCLC with a low or high PD‐L1 expression level, but not in those with intermediate PD‐L1 TPS with significant HR differences among the three subgroups of PD‐L1 status. Most of previous studies suggested that patients with NSCLC with a high PD‐L1 expression level had survival benefits from immune therapy rather than those with low or undetectable PD‐L1 status, but in a retrospective analysis of Phase III study (CheckMate 057) in patients with NSCLC treated with NIV or DOC, a small portion of subjects with PD‐L1 negative also experienced longer survival from ICI therapy (Borghaei et al., 2015). In the IMpower131 trial, compared with chemotherapy alone, although ATE plus PBC led to statically significant improvements in OS and PFS on the whole, the OS and PFS in the subgroups of PD‐L1 <1% or 1–49% did not have significance (Jotte et al., 2018). As previously discussed, the biological function of PD‐1 pathway appears to be more important than PD‐L1 status alone in forecasting the prognosis of patients with NSCLC treated with immunotherapy. Another possible explanation is that positive survival benefits for patients with PD‐L1 negative were observed from studies of CheckMate 017 (squamous‐cell NSCLC), KEYNOTE‐189 (nonsquamous‐cell NSCLC), and KEYNOTE‐407 (squamous‐cell NSCLC) studies (Brahmer et al., 2015; Gandhi et al., 2018; Paz‐Ares et al., 2018). What's more, CheckMate 227 was a Phase III study that aimed to evaluate the effects of ICI‐based treatment in patients with NSCLC with a high tumor mutational burden and revealed encouraging clinical results regardless of PD‐L1 status (PD‐L1 TPS <1% or ≥1%; Hellmann et al., 2018). In general, squamous patients with NSCLC bear a heavier tumor mutational burden with exception of targetable genomic mutations (e.g., EGFR, KRAS, or ROS1) than nonsquamous patients with NSCLC (Rizvi et al., 2015). Thus to some extent, for patients with NSCLC with negative PD‐L1 level, high tumor mutational burden increases the chance of tumor response to immunotherapy (Anagnostou et al., 2017; Rosenberg et al., 2016). Therefore, in this study, we concluded that PD‐L1 alone was not an ideal predictive biomarker for survival benefits of advanced patients with NSCLC from immunotherapy because of the unclear function and regulation of PD‐1 pathway and technical limitations including: archived or fresh tissue for PD‐L1 testing, optimal antibody (22C3, 28‐8, SP142, or SP263) and when to conduct PD‐L1 testing or whether to retest PD‐L1 expression (Brahmer et al., 2018; Shen & Zhao, 2018).

### 4.2 Strengthens and weaknesses of this study

First, we are the first to report the most comprehensive and the largest indirect analysis to our knowledge of the relative benefits and risks between ICI‐chemotherapy versus ICI‐monotherapy, especially that of PEM plus PBC versus PEM as monotherapy, and also indirectly compared the efficacy and safety of different ICI‐chemotherapy strategies, such as PEM plus PBC versus ATE plus PBC. As there is no comparison directly involved in the addition of chemotherapy to immunotherapy and immunotherapy alone, to some extent, this study bridged the gap between combining ICI therapy with standard‐of‐care chemotherapy. Our findings confirmed that ICI‐chemotherapy had a PFS advantage over ICI monotherapy with significant *p* value for interaction. Second, another distinct strength of this study was the quality of data included in this study. With information obtained from 20 well‐designed RCTs, we carried out quantitative analysis based on predefined primary endpoints of survival and second endpoints of TRAEs for more than 12,000 NSCLC patients, which has been the largest scale of NSCLC patients analyzed so far. In general, a large‐scale number of subjects involved in a meta‐analysis are crucial so as to reduce the occurrence of statistical errors. Third, our study recommended PEM plus PBC as the optimal therapeutic option for advanced patients with NSCLC with no actionable genetic mutations. Fourth, compared with the consensus statement of the SITC, our study took practice‐changing updates into account from another nine randomized trials with 6,070 subjects including CA184‐041, CA184‐104, CheckMate 078, IMpower130, IMpower132, IMpower150, JAVLIN LUNG 200, KEYNOTE‐042, and KEYNOTE‐407 (Brahmer et al., 2018; Shen & Zhao, 2018). Overall, the SITC conducted selection of NSCLC patient's selection mainly based on histology subgroup, PD‐L1 status (TPS ≥50% or TPS <50%), and genetic aberrations. These selection criteria aside, our study further selected patients on the basis of a more specific PD‐L1 level, which classified into TPS <1%, TPS 1–49%, and TPS ≥50%. So, our results could be said to be more convincing evidence of clinical practice with respect to determining eligible patients treated with immune checkpoint inhibitor therapy, especially for those with an intermediate PD‐L1 status.

Albeit the strengths above, the study has several limitations. One is that data were obtained from published articles or conference presentations, however, none of them were presented as individual patients' data. Thus, some potential variants (e.g., tumor mutational burden) were missed in our study, which might result in difference to our current findings regarding with the clinical activity of ICIs. Therefore, our results of subgroup analysis remains suggestive but not conclusive. Another limitation is that the differences of benefits and risks in subgroup of ICI‐chemotherapy versus ICI monotherapy did not come to conclusion through indirect analysis. To date, no RCTs have been designed to compare ICI‐chemotherapy directly with ICI as monotherapy for patients with NSCLC, so we conducted a cross study analysis with data from Phase III RCTs of high quality. However, these results should be interpreted with caution. Third, the indirect methods of comparison require that the enrolled RCTs should be comparable with respect to potential factors of therapeutic effects and the weak heterogeneity across the indirect comparisons indicated that our results were true. Fourth, the toxicity profile is as crucial as survival benefits to determine the optimal treatment choice for patients with NSCLC. Although overall, we took Grade 1–5 and Grade 3–5 TRAEs into account, we could not deal with the issue in the subgroups because data concerning TRAEs of involved populations stratified by PD‐L1 status were not available.

## 5 CONCLUSION

In conclusion, for advanced patients with NSCLC, ICI therapies with or without PBC are promising therapeutic options with advantageous survival, clinical response rate and a manageable safety profile than chemotherapy. Furthermore, PEM plus PBC is recommended as the optimal first‐line therapy option for patients with NSCLC without targetable genomic mutations. In addition, PD‐L1 alone is not recommended as an adequate molecular biomarker to identify eligible patients for routine clinical practice in immunotherapy.

## CONFLICT OF INTERESTS

The authors declare that there are no conflict of interests.

## AUTHOR CONTRIBUTIONS

W. L. and D. L. conceived and designed the study. C. W., W. Q., and Y. J collected the data, analyzed the data and wrote the manuscript. M. Z., J. S., and T.W. assisted with the data analyses and participated in the writing of manuscript. All authors read and approved the final manuscript.

## Supporting information

Supporting information

Supporting information

Supporting information

Supporting information

Supporting information

Supporting information

Supporting information

Supporting information

Supporting information

Supporting information

## Data Availability

All the data obtained and/or analyzed during the current study were available from the corresponding authors on reasonable request.
