# Supplementary material for: The landscape of immune checkpoint inhibitor plus chemotherapy versus immunotherapy for advanced non‐small‐cell lung cancer: A systematic review and meta‐analysis
Source: J Cell Physiol. 2019 Nov 6;235(5):4913–27. doi: 10.1002/jcp.29371 (PMC7028135; doi:10.1002/jcp.29371)
Supplement: Supplementary file 9 — Supporting information [file JCP-235-4913-s009.docx]

**Table S1. Search strategies (3,691 results)**

**PubMed**: 1,227 results

((“Nivolumab "[All Fields] OR "Opdivo"[All Fields] OR "ONO-4538"[All Fields] OR "MDX-1106"[All Fields] OR "BMS-936558"[All Fields] OR "Nivo"[All Fields] OR "Pembrolizumab"[Supplementary Concept] OR "Pembrolizumab"[All Fields] OR "lambrolizumab"[All Fields] OR "keytruda"[All Fields] OR "SCH 900475"[All Fields] OR "MK-3475"[All Fields]) OR "Nivolumab"[Supplementary Concept])) OR (("Avelumab"[Supplementary Concept] OR " Avelumab "[All Fields] OR "Atezolizumab"[Supplementary Concept] OR "Atezolizumab "[All Fields] OR "MSB0010718C"[All Fields] OR "Tecentriq"[All Fields] OR "RO5541267"[All Fields] OR "RG7446"[All Fields] OR "MPDL3280A"[All Fields])) OR ("Durvalumab"[Supplementary Concept] OR "Durvalumab "[All Fields] OR "MEDI-4736"[All Fields] OR "MEDI4736"[All Fields])) OR)) OR ("Ipilimumab"[Supplementary Concept] OR "Ipilimumab"[All Fields] OR "MDX-CTLA-4"[All Fields] OR "Yervoy"[All Fields])) OR ("Tremelimumab"[Supplementary Concept] OR " Tremelimumab"[All Fields])) OR ("programmed cell death-1"[All Fields] OR "PD-1 "[All Fields] OR "programmed cell death ligand-1"[All Fields] OR "PD-L1"[All Fields] OR "cytotoxic T-lymphocyte associated antigen-4"[All Fields] OR "CTLA-4"[All Fields] OR "immune checkpoint inhibitor"[All Field]))AND ("lung carcinoma"[ All Fields] OR "pulmonary carcinoma"[ All Fields] OR ("lung cancer"[All Fields] OR "lung tumor"[All Fields] OR "NSCLC"[Title/Abstract])) AND ("clinical trials as topic"[MeSH Terms] OR "trial"[Title/Abstract] OR "study"[Title/Abstract] OR "randomized controlled trial"[Title/Abstract]))

**Embase**:1,718 results

('Nivolumab' OR 'Opdivo' OR 'ONO-4538' OR 'MDX-1106' OR 'BMS-936558' OR 'Nivo' OR 'Pembrolizumab' OR 'lambrolizumab' OR 'keytruda' OR 'SCH 900475' OR 'MK- 3475' OR 'Atezolizumab' OR 'Tecentriq' OR 'Durvalumab' OR 'MEDI-4736' OR 'MEDI4736' OR 'Avelumab' OR ' Ipilimumab' OR 'MDX-CTLA-4' OR ' Yervoy' OR 'Tremelimumab' OR 'immiune checkpoint inhibitor' OR 'programmed cell death-1' OR 'PD-1' OR ' programmed cell death ligand-1' OR 'PD-L1' OR 'cytotoxic T-lymphocyte associated antigen-4' OR 'CTLA-4') AND ('lung cancer' OR 'lung tumor' OR 'NSCLC' OR 'lung carcinoma' OR 'pulmonary carcinoma') AND ('randomized controlled trial' or 'controlled clinical trial') AND ‘human’

**Cochrane**: 746 results

('Pembrolizumab' OR 'lambrolizumab' OR 'keytruda' OR 'SCH 900475' OR 'MK- 3475' OR 'Nivolumab' OR 'Opdivo' OR 'ONO-4538' OR 'MDX-1106' OR 'BMS-936558' OR 'Nivo' OR ' Atezolizumab' OR 'MSB0010718C' OR 'Tecentriq' OR 'RO5541267' OR 'RG7446' OR 'MPDL3280A' OR 'Durvalumab' OR 'MEDI-4736' OR 'MEDI4736' OR 'Avelumab' OR ' Ipilimumab ' OR 'Tremelimumab' OR 'checkpoint inhibitor' OR 'programmed cell death-1' OR 'PD-1' OR ' programmed cell death ligand-1' OR 'PD-L1' OR 'cytotoxic T-lymphocyte associated antigen-4' OR 'CTLA-4') AND ('lung carcinoma' OR 'pulmonary carcinoma' OR 'lung cancer' OR 'lung tumor' OR 'NSCLC' ) AND ( 'trials')

**Table S2. Quality assessment: risk of bias by Cochrane Collaboration’s tool.**

| **Study ID** | **Year** | **Sequence generation** | **Allocation concealment** | **Blinding** | **Incomplete outcome data** | **Selective reporting** | **Other source of bias** |
| --- | --- | --- | --- | --- | --- | --- | --- |
| CheckMate 017 | 2015 | Low Risk | High Risk | High Risk | Low Risk | Low Risk |  |
| CheckMate 026 | 2017 | Low Risk | Unclear Risk | High Risk | Low Risk | Low Risk |  |
| CheckMate 057 | 2015 | Low Risk | High Risk | High Risk | Low Risk | Low Risk |  |
| CheckMate 078 | 2019 | Low Risk | High Risk | High Risk | Low Risk | Low Risk |  |
| JAVELIN Lung 200 | 2018 | Low Risk | High Risk | High Risk | Low Risk | Low Risk |  |
| KEYNOTE-010(a) | 2016 | Low Risk | Low Risk | High Risk | Low Risk | Low Risk |  |
| KEYNOTE-010(b) | 2016 | Low Risk | Low Risk | High Risk | Low Risk | Low Risk |  |
| KEYNOTE-024 | 2016 | Low Risk | Unclear Risk | High Risk | Low Risk | Low Risk |  |
| KETNOTE-042 | 2019 | Low Risk | Unclear Risk | High Risk | Low Risk | Low Risk |  |
| OAK | 2017 | Low Risk | High Risk | High Risk | Low Risk | Low Risk |  |
| POPLAR | 2016 | Low Risk | High Risk | High Risk | Low Risk | Low Risk |  |
| CA184-041 | 2012 | Unclear Risk | Unclear Risk | Low Risk | Low Risk | Low Risk |  |
| CA184-104 | 2017 | Low Risk | Unclear Risk | Low Risk | Low Risk | Low Risk |  |
| CheckMate 227(a) | 2018 | Low Risk | Low Risk | Low Risk | Low Risk | High Risk |  |
| CheckMate 227(b) | 2018 | Low Risk | Low Risk | Low Risk | High Risk | High Risk | Data from the abstract and the presentation slides |
| IMpower130 | 2019 | Low Risk | Low Risk | Low Risk | Low Risk | Low Risk |  |
| IMpower131 | 2018 | Low Risk | Low Risk | High Risk | Low Risk | Low Risk | Data from the abstract and the presentation slides |
| IMpower132 | 2018 | Low Risk | Low Risk | High Risk | Low Risk | Low Risk | Data from the abstract and the presentation slides |
| IMpower150 | 2018 | Low Risk | Low Risk | Low Risk | Low Risk | Low Risk |  |
| KEYNOTE-021 | 2016 | Low Risk | Low Risk | Low Risk | Low Risk | Low Risk |  |
| KEYNOTE-189 | 2018 | Low Risk | Low Risk | Low Risk | Low Risk | Low Risk |  |
| KEYNOTE-407 | 2018 | Low Risk | Low Risk | Low Risk | Low Risk | Low Risk |  |

**Table S3. Differences in OS benefits of Immunotherapy in ICI-chemotherapy and ICI alone by subgroups.**

| **Variable** | **Study** | **Pooled HR (95%CI)** | | **Test for Difference** | |
| --- | --- | --- | --- | --- | --- |
|  |  | **ICI-Chemotherapy** | **ICI alone** | **I2,%** | **P Value** |
| **Overall** | 20 | 0.77 [0.66; 0.89] | 0.74 [0.68; 0.82] | 0 | 0.74 |
| **Sex** |  |  |  |  |  |
| Male | 13 | 0.80 [0.71; 0.91] | 0.75 [0.69; 0.82] | 0 | 0.42 |
| Female | 13 | 0.58 [0.30; 1.10] | 0.83 [0.71; 0.96] | 12 | 0.29 |
| **Age** |  |  |  |  |  |
| ≥ 65 yr | 12 | 0.84 [0.72; 0.98] | 0.79 [0.71; 0.88] | 0 | 0.54 |
| < 65 yr | 12 | 0.63 [0.46; 0.86] | 0.78 [0.67; 0.90] | 30 | 0.23 |
| **Smoking status** |  |  |  |  |  |
| Nonsmoker | 7 | 0.36 [0.15; 0.85] | 1.01 [0.79; 1.29] | 80 | 0.02 |
| Ever smoker | 9 | 0.74 [0.56; 0.97] | 0.78 [0.68; 0.90] | 0 | 0.70 |
| **First line** | 12 | 0.78 [0.68; 0.90] | 0.83 [0.64; 1.08] | 0 | 0.70 |
| **Histological type** |  |  |  |  |  |
| Squamous | 9 | 0.87 [0.77; 0.98] | 0.71 [0.63; 0.81] | 15 | 0.31 |
| Non-squamous | 11 | 0.69 [0.57; 0.85] | 0.83 [0.70; 0.98] | 44 | 0.18 |
| **Class of immunotherapy** |  |  |  |  |  |
| Anti-PD-1 | 11 | 0.56 [0.47; 0.67] | 0.72 [0.64; 0.82] | 82 | 0.02 |
| Anti-PD-L1 | 7 | 0.83 [0.75; 0.93] | 0.81 [0.72; 0.90] | 0 | 0.67 |
| **PD-L1 Status** |  |  |  |  |  |
| < 1% | 10 | 0.78 [0.67; 0.90] | 0.78 [0.67; 0.91] | 0 | 0.54 |
| 1 - 49% | 9 | 0.77 [0.55; 1.07] | 0.92 [0.76; 1.12] | 0 | 0.36 |
| ≥ 50% | 13 | 0.61 [0.49; 0.77] | 0.63 [0.56; 0.71] | 32 | 0.14 |
| **ECOG PS** |  |  |  |  |  |
| 0 | 11 | 0.77 [0.63; 0.94] | 0.76 [0.66; 0.87] | 43 | 0.07 |
| 1 | 11 | 0.77 [0.70; 0.85] | 0.76 [0.69; 0.83] | 66 | 0.002 |
