# Supplementary material for: The landscape of immune checkpoint inhibitor plus chemotherapy versus immunotherapy for advanced non‐small‐cell lung cancer: A systematic review and meta‐analysis
Source: J Cell Physiol. 2019 Nov 6;235(5):4913–27. doi: 10.1002/jcp.29371 (PMC7028135; doi:10.1002/jcp.29371)
Supplement: Supplementary file 10 — Supporting information [file JCP-235-4913-s010.docx]

Figure S1. Study selection procedure.

Figure S2. Sensitivity analyses of overall survival (left) and progression-free survival (right) by repeating the pooled analyses with one study omitted at a time.

Figure S3. Sensitivity analyses of overall survival (left) and progression-free survival (right) after removing studies that were only available from conference proceedings by repeating the pooled analyses with one study omitted at a time.

Figure S4. Publication bias comparing hazard ratios (HR) for overall survival (left) and progression-free survival (right).

Figure S5. Forest plots of hazard ratios comparing progression-free survival between ICI therapies and chemotherapy according to PD-L1status.

Figure S6. Subgroup analysis of hazard ratios and ratio ratios comparing overall survival (OS), progression-free survival (PFS), objective response rate (ORR), treatment-related adverse events of any grade (Grade 1-5 TRAEs) and treatment-related adverse events of grade 3-5 (Grade 3-5 TRAEs) in patients who received ICI therapies to chemotherapy in NSCLC.

Figure S7. Overall indirect comparisons of hazard ratios between pembrolizumab plus platinum-based chemotherapy versus pembrolizumab alone; indirect analysis of overall survival (OS) and progression-free survival (PFS) based on histology type and PD-L1 status.

Table S1. Search strategies.

Table S2. Quality assessment: risk of bias by Cochrane Collaboration’s tool.

Table S3. Differences in OS benefits of ICI-chemotherapy and ICI monotherapy by subgroups.
